# Lifespan Regulation by Evolutionarily Conserved Genes Essential for Viability

**DOI:** 10.1371/journal.pgen.0030056

**Published:** 2007-04-06

**Authors:** Sean P Curran, Gary Ruvkun

**Affiliations:** 1 Department of Molecular Biology, Massachusetts General Hospital, Boston, Massachusetts, United States of America; 2 Department of Genetics, Harvard Medical School, Boston, Massachusetts, United States of America; Stanford University School of Medicine, United States of America

## Abstract

Evolutionarily conserved mechanisms that control aging are predicted to have prereproductive functions in order to be subject to natural selection. Genes that are essential for growth and development are highly conserved in evolution, but their role in longevity has not previously been assessed. We screened 2,700 genes essential for Caenorhabditis elegans development and identified 64 genes that extend lifespan when inactivated postdevelopmentally. These candidate lifespan regulators are highly conserved from yeast to humans. Classification of the candidate lifespan regulators into functional groups identified the expected insulin and metabolic pathways but also revealed enrichment for translation, RNA, and chromatin factors. Many of these essential gene inactivations extend lifespan as much as the strongest known regulators of aging. Early gene inactivations of these essential genes caused growth arrest at larval stages, and some of these arrested animals live much longer than wild-type adults. *daf-16* is required for the enhanced survival of arrested larvae, suggesting that the increased longevity is a physiological response to the essential gene inactivation. These results suggest that insulin-signaling pathways play a role in regulation of aging at any stage in life.

## Author Summary

The lifespan of an animal is determined by both environmental and genetic factors, and many of the mechanisms identified to increase lifespan are evolutionarily conserved across organisms. Previous longevity screens in C. elegans have identified over 100 genes, but ∼2,700 essential for normal development were excluded from analysis. Paradoxically, these essential genes are five times more likely to be highly conserved in phylogeny than genes with no obvious developmental phenotypes. We screened these 2,700 essential genes for increased adult lifespan by initiating the gene knockdown once the animal had reached adulthood, thus bypassing earlier developmental roles. We identified 64 genes that can extend lifespan when inactivated postdevelopmentally. More than 90% of the genes we identified are conserved from yeast to humans. Many of the newly identified longevity genes extend lifespan as robustly as the most well-characterized longevity mutants. It is possible that the homologues of these genes may also regulate lifespan in other organisms as well. Genetic analysis places some of these genes in known pathways regulated by insulin-like signaling, although many of these gene inactivations function independently of this mechanism of lifespan extension. Surprisingly, a subset of these gene inactivations that induce potent developmental arrest also facilitate enhanced survival in the arrested state, suggesting that aging at any stage may be subject to regulatory control.

## Introduction

The lifespan of an organism is regulated by both genetic and environmental influences in many species [1]. Recent work has identified specific components from a variety of cellular processes that regulate lifespan. In C. elegans loss-of-function mutations in the insulin/insulin-like growth factor-1/*daf-2* signaling pathway can more than double the lifespan of an animal [2–6]. The regulation of lifespan by DAF-2 occurs during adulthood [7]. The insulin-signaling pathway negatively regulates the forkhead (FOXO) transcription factor DAF-16, which ultimately functions to both positively and negatively regulate transcription of metabolic, chaperone, cellular defense, and other genes [8–11]. The regulation of lifespan through an insulin-like signaling cascade is an evolutionarily conserved mechanism and has been demonstrated in flies and mice [12–15]. The regulation of DAF-16 activity is also modulated by the JNK signaling pathway, the SIR-2.1 deacetylase, and HSF-1, LIN-14, and SMK-1 in the nucleus [16–20].

In many organisms the rate of aging is tied to reproduction. In C. elegans germline proliferation produces a DAF-16 and KRI-1 mediated signal that negatively regulates lifespan while the somatic gonad promotes lifespan extension [21–23].

Caloric restriction (CR) also extends lifespan across species including yeast, worms, flies, and mice [24–29]. The *sir-2.1* and *let-363* genes in C. elegans regulate lifespan via CR [30,31]. Unlike insulin signaling, in flies, imposing CR at anytime can increase lifespan [32]. Finally, perturbations in mitochondrial function have been shown to increase lifespan [33–36]. Diminished function of the mitochondrial electron transport system can further extend mutations in the insulin-like signaling pathway, however mutations in the ubiquinone biosynthesis gene *clk-1* do not further increase the longevity phenotype from CR. The mechanism of longevity induced by defective mitochondria is thought to occur during development, as previous attempts to use RNA interference (RNAi) to inhibit mitochondrial function in adults has not been shown to increase lifespan [34].

Recent genome-wide RNAi screens for increased longevity have identified ∼100 potential regulators of lifespan in C. elegans from diverse cellular pathways, many of which are evolutionarily conserved [37,38], but genes essential for viability are underrepresented in genome-wide RNAi screens for postdevelopmental phenotypes such as aging. These RNAi screens for adult longevity preclude the identification of gene inactivations that cause lethality, larval arrest, sterility, and/or other developmental pleiotropies (i.e., essential genes), unless these genes are inactivated after their required developmental roles.

Here we report 64 genes that, when inactivated postdevelopmentally by RNAi, increase adult lifespan. We also report the enhanced survival phenotype of the animals arrested during development by these essential gene inactivations.

## Results/Discussion

### Postdevelopmental *RNAi* Screen for Clones That Increase Lifespan

**Figure 1:**
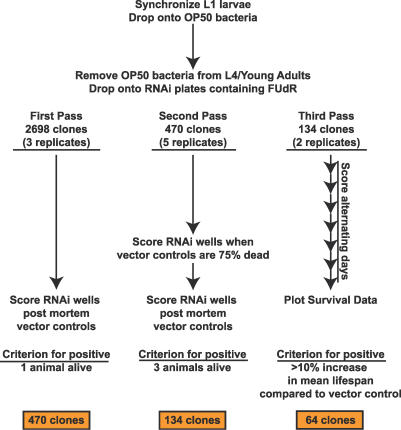
Representation of the Postdevelopmental RNAi Screen for Longevity Embryos from gravid C. elegans hermaphrodites were isolated by hypochlorite treatment and allowed to hatch overnight in the absence of food. The synchronized L1 larvae were then placed on OP50 agar plates at 20 °C and allowed to develop to L4/young adult stage. The L4/young adult animals were then washed from the plates, cleaned from bacteria by sucrose flotation, and placed on six-well RNAi plates (∼30 worms/well). From three replicates of the first pass, 470 RNAi clones were identified as positive and were retested in the second pass under similar conditions while increasing the stringency for scoring positive. A total of 134 RNAi clones passed the second pass criteria and were scored longitudinally in the third pass against blind positive (*daf-2* RNAi) and negative (empty vector, *eri-1* RNAi, and *daf-16* RNAi) controls. A total of 64 RNAi clones increased mean lifespan by >10% compared to the negative controls.

To identify essential genes that function in adulthood to regulate lifespan, we selected 2,700 RNAi clones that, if fed from the previous generation or from the L1 larval stage, cause arrest at embryonic or larval stages, and screened them for increased lifespan after initiating RNAi at the L4 larval/young adult stage (Figure 1). We found three observations that validate this approach: First, C. elegans lifespan can be extended when fed dsRNA targeting the insulin receptor/*daf-2* at any developmental larval stage through adulthood [7]; second, conditional *daf-2* alleles cause dauer arrest if the gene is inactivated at the L1 stage but increased longevity if inactivated in adults [39]; and third, null alleles of *daf-2* are lethal [40] demonstrating the necessity of uncoupling the developmental and aging phenotypes of essential genes as well as the utility of producing non-null phenotypes by RNAi.

We performed the RNAi screen utilizing the *eri-1(mg366)* [41] strain to improve RNAi in all cells types including neurons, which are normally refractory to RNA interference. In fact, many of the longevity genes we identify are expressed in the nervous system, which has been implicated in insulin regulation of longevity (Figure S1; Table S1) [42].

Gene inactivations that caused increases in mean lifespan of at least 10% were scored as positive in our screen. Endocrine signaling, stress adaptation, metabolism, and reproduction are potent and evolutionarily conserved regulators of aging [43]. Our screen identified genes from these canonical longevity-promoting pathways in addition to pathways not previously implicated in aging (Figures S2 and S3; Table 1).

**Table 1:**
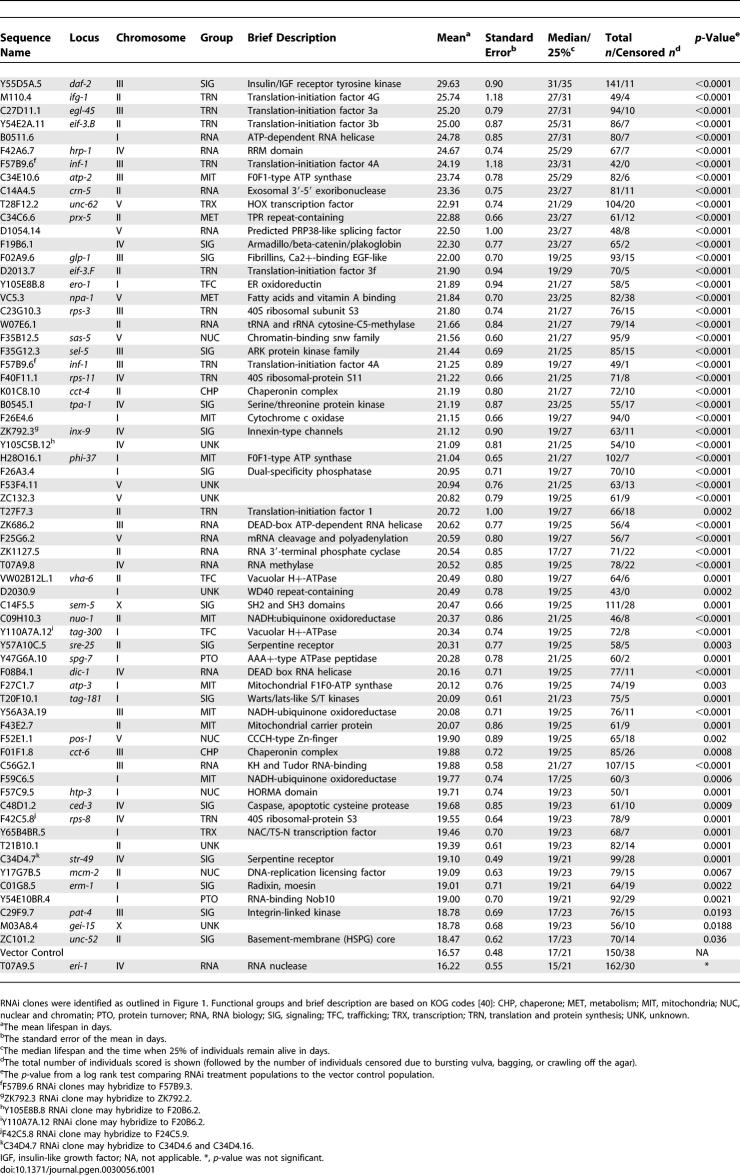
64 RNAi Clones That Increase Adult Lifespan When Fed Postdevelopmentally

Essential genes are more conserved in phylogeny than genes with no obvious developmental phenotype; more than 90% of the genes we identify are conserved from yeast to humans. The theory of antagonistic pleiotropy suggests that any genes that function in postreproductive longevity control should be under natural selection at prereproductive stages. Our data support this notion: our yield of major lifespan regulators is 64 gene inactivations out of 2,700 tested (∼2.4%), a 4-fold increased yield compared to the previous 89 gene inactivations out of 16,000 screened (∼0.6%), and a higher proportion of the gene inactivations cause large increases in longevity (percent increase compared to vector control denoted in parentheses for each RNAi clone), although the use of an enhanced RNAi strain may also contribute to the increased sensitivity.

### Functional Classes of Postdevelopmental Regulators of Longevity

#### Protein synthesis.

Sorting by the lifespan increase, inactivation of genes involved in protein synthesis caused the most potent lifespan increase. We identified several RNAi clones that target components of the translation initiation factor (eIF) complex; *egl-45* (eIF3, 52%), *eif-3.F* (eIF3, 32%), *eif-3.B* (eIF3, 51%), *ifg-1* (eIF4G, 55%), *T27F7.3* (eIF1, 25%), and two clones targeting *inf-1* (eIF4A, 46% and 28%). Inhibition of these genes increased lifespan up to 50% longer than with vector control RNAi. In mammals, insulin stimulates protein synthesis in vivo and in vitro by increasing the rate of translation initiation by regulating the eIF4F complex consisting of eIF4G, eIF4E, eIF4A, and the 40S ribosome [44]. eIF4F then recruits eIF3 to generate the 43S ribosome. Protein synthesis may be closely monitored in the cell to allow energy equivalents to be redirected to other processes such as genomic maintenance and stress adaptation. Mice lacking the translational inhibitor 4E-BP1 are lean and have increased metabolism and mitochondrial biogenesis, which are opposing phenotypes found in long-lived mutants [45]. We found that three clones that target components of the 40S subunit of the ribosome, *rps-3* (32%), *rps-8* (18%), and *rps-11* (28%) also increased mean lifespan. Although components of the 80S ribosome complex were represented in the RNAi library that we screened, only components of the 43S complex, predominantly translation initiation factors emerged as regulators of lifespan.

Under conditions of ER stress a transient block in protein synthesis occurs. Inactivation of the ER oxidoreductase *ero-1* (32%) also increased lifespan (Table 1). In mammals*, C. elegans,* and yeast, the ATF4/*atf-5*/GCN4 gene contains an extensive 5′UTR with multiple upstream open reading frames. Under normal conditions the upstream open reading frames are preferentially utilized for translation initiation yielding truncated proteins, whereas under low nutrient conditions the proper ATG is used to synthesize ATF4/*atf-5*/GCN4. eIF1 has been shown to regulate initiation codon selection [46]. In support of our findings two recent studies also identified *ifg-1* and *rps-11* as regulators of longevity [47,48]. Therefore, although complex, translation can be an effective regulator of cellular stress and adaptation.

#### Signaling.

Genes in the insulin-signaling pathway are well-established and potent regulators of longevity in the worm [43]. Our screen identified many signaling molecules that extend adult lifespan (Figure S2). For example, we identify SEM-5 (24%), a SH2- and SH3-like protein that negatively regulates RAS-MAP and -IP3 signal transduction. *sem-5* was also identified as an enhancer of dauer formation (S.S. Lee, unpublished data). In addition, inactivation of four annotated kinases—the serine/threonine protein kinases *tpa-1* (28%) and *tag-181* (21%), an ARK kinase family member *sel-5* (29%), and a previously identified integrin-linked kinase *pat-4* (13%) [38]—increased lifespan. *daf-16(mgDf47)* epistasis (discussed below) reveals that some of these kinases may act in the insulin pathway.

**Figure 2:**
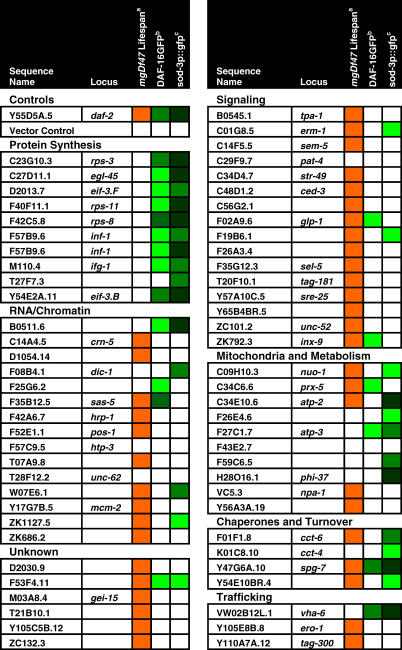
Identification of RNAi Clones that Act through DAF-16 (A) Shading indicates that *daf-16(mgDf47); eri-1(mg366)* is epistatic to the lifespan extension observed in *eri-1(mg366)* (Table S2). (B) DAF-16 nuclear localization after feeding RNAi clone is shown. Light shading indicates some nuclear localization, and medium shading indicates more nuclear localization (Figure S4). (C) *sod-3p::gfp* expression after RNAi feeding is shown. Light shading indicates weak expression, medium shading indicated modest expression, and dark shading indicates strong expression.

The C. elegans genome contains one insulin-like receptor, *daf-2*, but 39 insulin-like peptides of which only a few have known functions [49]. Loss-of-function mutations in *daf-2* are of the strongest enhancers of lifespan in *C. elegans,* and in our screen RNAi of *daf-2* increased lifespan by ∼79%. In humans, the relaxin family of peptides are structurally similar to insulin and bind to seven transmembrane receptors to initiate signaling cascades [50]. We identified two serpentine receptors, *str-49* (15%) and *sre-25* (23%), that act in lifespan control*.* Since the lifespan extension phenotype of these gene inactivations requires DAF-16 (Figure 2), it is conceivable that these seven transmembrane receptors utilize a subset of the insulin-like peptides, in analogy to relaxin in humans**.**

Signals from proliferating germ cells negatively regulate C. elegans aging, via the insulin-signaling pathway [43]. We identified *glp-1* (33%), which codes for a DSL-family ligand receptor in the germline that controls germ cell proliferation and negatively regulates lifespan [22]. The design of our screen was fortunate in that the somatic gonad and the establishment of the initial pool of proliferating germ cells at adulthood was not perturbed. Down-regulating genes such as *glp-1* or genes that function in the reproductive system (Table S1) could disrupt signaling from the germline to increase lifespan.

Vacuolar H^+^-ATPases were another potent lifespan regulator to emerge from the screen. These proteins acidify intracellular compartments and act in synaptic transmission and cell death signaling cascades [51]. UNC-32, a subunit of the vacuolar ATPase, regulates male longevity [52]. The vacuolar ATPase is comprised of a V0/V1 heteromultimer. We identified one of each type of subunit from our screen; *vha-6* (V0, 24%) and *tag-300* (V1, 23%). In addition, we identified two other candidate longevity genes linked to cell-death pathways; the apoptotic cysteine protease *ced-3* (19%) and the cell death related nuclease *crn-5* (41%). We were surprised to identify an RNAi clone targeting *ced-3* from our screen since *ced-3(0)* mutants fail to undergo programmed cell death but are not lethal [53]. However, we noted synthetic lethality between *ced-3(717)* and *daf-2(e1370)* indicating a genetic interaction between these longevity-promoting pathways (unpublished data). In support of this finding, the increased lifespan phenotype of *ced-3* (RNAi) was dependent upon *daf-16* (Figure 2).

#### Mitochondria.

We were surprised to find that late inactivation of mitochondrial genes also increased lifespan. RNAi clones targeting *F26E4.6* (28%) and *atp-3* (21%) were previously identified to increase lifespan [37,38]. However, previous work has suggested that perturbations that disable mitochondria to increase lifespan must occur during larval development, rather than after the L4 stage as we observe here [34]; these differences may be attributed to the enhanced RNA interference phenotype of the *eri-1(mg366)* strain. In addition, we identified seven other mitochondrial RNAi clones that significantly increased adult lifespan when the gene inactivation occurred postdevelopmentally: two subunits of respiratory complex V (*atp-2* [43%] and *phi-37* [27%]); three subunits of the NADH:ubiquinone oxidoreductase complex (*F59C6.5* [19%], *nuo-1* [23%], and *Y56A3A.19* [21%]); a predicted mitochondrial carrier protein (*F43E2.7*, 21%); and a mitochondrial AAA-protease *(spg-7*, 22%). In support of a role for *nuo-1* in negative regulation of lifespan, overexpression of *nuo-1* causes decreased fecundity and shortened lifespan [40].

#### Gene expression.

RNA binding/processing and chromatin-associated factors were among the largest class of regulators; comprising over 20% of the genes identified in our screen and yielding some of the most potent extension of lifespan (Figures S2 and S3; Table 1). The strong enrichment for RNA factors was intriguing because few have been identified in the previous genome-wide RNAi screens for longevity [37,38]. We identified three clones targeting predicted RNA helicases, *dic-1* (22%), *B0511.6* (50%), and *ZK686.2* (24%) that scored as among the strongest gene inactivations that increase lifespan (Table 1). We also found two clones with predicted conserved RNA binding domains; *hrp-1* (49%) contains an RRM domain, and *C56G2.1* (20%) has a KH and a Tudor domain that may facilitate RNA and chromatin interactions, respectively. In addition, the protein products of a large number of genes identified are predicted to modify and/or process RNA; these include a predicted mRNA splicing factor *D1054.14* (36%); two ribonucleases *crn-5* (41%) and *Y54E10BR.4* (15%); mRNA cleavage and polyadenylation factor *F25C6.2* (24%); RNA cyclase *ZK1127.5* (24%); and two RNA methylases, *T07A9.8* (24%) and *W07E6.1* (31%).

Chromatin-associated factors are conserved players in altering gene expression, coordinating cell fate, and modulating small RNA mechanisms of gene regulation in eukaryotes [54–56]. Inactivation of these chromatin-associated factors caused increased lifespan: *C56G2.1* (20%), *sas-5* (30%), *htp-3* (19%)*,* and the DNA replication licensing factor *mcm-2* (15%). In support of our findings, *mcm-2* was also identified by Serial Analysis of Gene Expression analysis to be transcriptionally repressed in *daf-2*/insulin-receptor mutants and may contribute to their longevity phenotype [57].

Two transcription factors, *unc-62/*homothorax/MEIS (38%) and *Y65B4BR.5*/NAC/TS-N (17%) were found to negatively regulate lifespan. Recently, the Dubcovsky laboratory reported that mutations in the NAC transcription factor *NAM-B1* delay senescence by more than three weeks in *Triticum turgidum,* and the ancestral wild wheat allele functions to accelerate senescence and increase nutrient remobilization [58], suggesting this may be an evolutionary conserved mechanism regulating lifespan. Although a role for *unc-62* has not previously been described in an adult animal, the *unc-62* promoter element is active in adult neuronal tissues (Figure S1). While a mechanism for RNA and chromatin-associated factors in controlling lifespan is unknown, there are significant alterations in the transcriptional profile of aging populations [59]; these genes may regulate such changes.

### Phenotypic Analysis of Candidate Longevity Genes

One established method of validating genes that emerge from RNAi screens is to test gene knockouts for the same phenotype. For these essential genes, it may prove possible to study the longevity of the arrested animals. However, to study adult longevity conditional alleles will be required. Such alleles tend to emerge from detailed genetic analysis rather than genomic knockout projects and are not currently available.

The aging research community has characterized several central mechanisms that mediate lifespan regulation including insulin/insulin-like growth factor signaling, CR, and mitochondrial function. To classify the pathways represented by these new genes, we performed secondary assays: DAF-16 localization, *sod-3* expression, arrested larval survival, suppression of polyglutamine aggregation, and aberrant fat metabolism and clustered the genes by the phenotypes observed (Figure 2; Figure S4; Table 2; Tables S1 and S2).

**Table 2:**
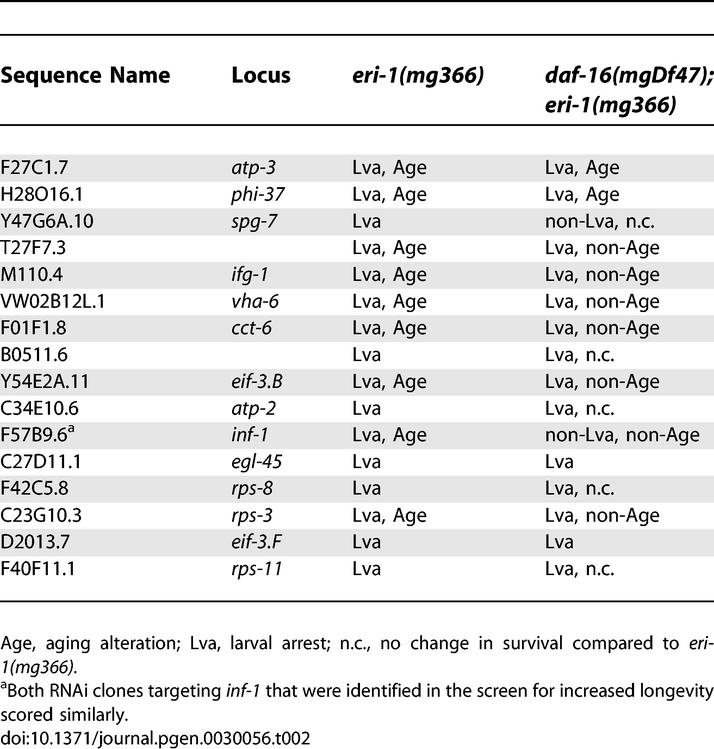
Larval Arrest and Survival Phenotypes of RNAi Clones during Development

#### Identification of clones that act in or converge upon the insulin-signaling pathway.

The DAF-16/FOXO transcription factor is a key player in many biological processes that regulate lifespan including stress adaptation and metabolism [10,11,39,60]. Because loss of function *daf-16* alleles are epistatic to many longevity promoting mutations, we inactivated the 64 candidate longevity genes in an *eri-1(mg366)* strain also carrying a null *daf-16(mgDf47)* mutation and scored for lifespan extension (Figure 2; Table S2). In accord with previous studies, most RNAi clones targeting mitochondrial genes that increase adult lifespan did not depend on DAF-16 [34,35]. The gene inactivations targeting the protein synthesis machinery also increase lifespan in the absence of DAF-16. However, RNAi of a few representative genes from most classes could increase the lifespan of the *daf-16(mgDf47)*;*eri-1(mg366)* strain and thus represent DAF-16 independent pathways of lifespan extension (Figure 2). We classified the remaining RNAi clones as DAF-16 dependent because they failed to extend mean lifespan in the absence of DAF-16 by at least 10% compared to vector controls. Among the DAF-16-dependent genes were most of the signaling molecules and RNA-related genes including *mcm-2, hrp-1, pos-1, sas-5, crn-5, T07A9.8, ZK686.2,* and *ZK1127.5*. These genes may act in or converge onto the insulin-signaling pathway for lifespan control.

Although the role of DAF-16 in insulin-signaling is the best understood, DAF-16 receives inputs from multiple cellular pathways and has functions outside of adult lifespan extension. DAF-16 transcriptional activity is negatively regulated by cytoplasmic sequestration. Upon loss of inhibition, DAF-16 translocates to the nucleus. Therefore, we tested whether any of the gene inactivations identified in our screen could induce DAF-16 nuclear localization, regardless of the necessity for DAF-16 in adult lifespan extension. Despite being independent of *daf-16* gene activity for increasing adult lifespan, inactivation of *atp-3, ifg-1, vha-6, F25G6.2,* *B0511.6**, eif-3.B, inf-1, egl-45, rps-8, rps-3, eif-3.F,* or *rps-11* induced localization of DAF-16 to the nucleus and consequently drove expression of the DAF-16 target gene *sod-3* (Figure 2). Since these genes do not function through DAF-16 to extend lifespan, the translocation of DAF-16 to the nucleus may be due to a general stress or may represent a new regulator of DAF-16 activity.

#### Larval arrest and survival phenotypes of RNAi clones during development.

Loss of DAF-2 during development causes constitutive arrest in the dauer stage. *daf-16(lf)* mutations are defective for dauer arrest induced by low insulin signaling and also fail to arrest as L1 stage larvae in the absence of food [60,61]. Many of the 2,700 gene inactivations initiated by feeding RNAi from the L1 stage cause highly penetrant developmental arrest at stages ranging from early larval to sterile adult stage (Table S1). A subset of these gene inactivations caused arrested larvae to survive longer than control animals and facilitated DAF-16 nuclear localization at the arrested stage (Figure 2; Table 2). We tested if the larval arrest and increased survival of the arrested larvae induced by these gene inactivations depend on DAF-16 activity by feeding the RNAi clones that cause larval arrest in *eri-1(mg366)* to a *daf-16(mgDf47);eri-1(mg366)* double mutant. The absence of DAF-16 abrogated the long-lived phenotype for most of the arrested larvae (Table 2). For most gene inactivations, *daf-16* mutant animals still arrested when fed the RNAi clone; however they did not survive as long in the arrested state. The *daf-16(mgDf47)* allele did, however, weakly suppress the larval arrest of *inf-1* or *spg-7* inactivation (Table 2). These data suggest that at almost any stage, stress pathways requiring *daf-16* may be triggered by gene inactivations to ensure the survival of the arrested larvae.

We noted that many of the gene inactivations that cause increased survival of arrested larvae encode translation factors. Translation is a major target of antibiotics, which are produced by a wide range of fungi and microbes that nematodes encounter in the environment. As a larvae or adult enters an environment with an antibiotic, there may be signaling pathways that detect ribosomal deficiency to trigger developmental arrest as well as xenobiotic protective pathways. The induced stress adaptation and survival pathways would ensure that the animal could live long enough to escape the antibiotic and resume reproductive development. Inhibition of translation by RNAi of ribosomal and other translation factors may mimic the ribosomal deficiencies induced by antibiotics in the normal C. elegans ecosystem, and trigger this physiological response, developmental arrest, and cessation of aging. Because it is a prereproductive arrest, this response may be under natural selection to trigger longevity enhancing pathways [62]. It may be significant that other gene inactivations with potent arrested larval lifespan increases, a vacuolar ATPase and the mitochondrial ATP synthase, are also targets of natural antibiotics [63,64].

### Summary

In this study, we have screened a large portion of the genome previously underrepresented in genome-wide based RNAi screens for aging phenotypes and identified 64 genes that normally function to shorten lifespan. Characterization of these gene knockdowns by epistasis with the insulin-signaling pathway and use of biomarkers of aging places them into distinct classes. Because these molecules are predicted to function in complex cellular pathways (Figure S5), future work will focus to dissect the mechanisms employed by these essential processes to regulate lifespan.

## Materials and Methods

### Strains.

Strains were maintained and cultured using standard techniques [65]. We used the following C. elegans strains and mutant alleles: wild-type N2 Bristol, *eri-1(mg366)IV, daf-16(mgDf47)I;eri-1(mg366)IV;* zIs356, *(daf-16p::daf-16::gfp; rol-6[su1006]);* muIs84, *(sod-3p::gfp);* huIs33, *(sod-3p::gfp; rol-6[su1006]);* and rmIs133: *(unc-54p::Q40yfp)*.

### Postdevelopmental RNAi lifespan screen.

Eggs were isolated from gravid *eri-1(mg366)* worms and synchronized by hatching overnight in the absence of food. The synchronized L1 larvae were then placed on OP50-containing agar plates and allowed to develop to L4-stage larvae at 20 °C. The L4-stage larvae were washed thoroughly, cleaned by sucrose flotation, and placed on 12-well plates with Escherichia coli expressing double-stranded RNA (dsRNA) (described below). We carried out a large-scale RNAi screen using the enhanced RNAi strain *eri-1(mg366)* (Figure S1). Briefly, each RNAi colony was grown overnight in LB with 50 μg/ml ampicillin and then seeded onto 12-well RNAi agar plates containing 5 mM isopropylthiogalactoside (IPTG). The RNAi bacteria were induced overnight at room temperature for dsRNA expression. We then added ∼30 synchronized L4-stage animals to each well, allowed worms to develop to adults, and then added 5-fluorodexoyuridine (FUdR) solution to a final concentration of 0.1 mg/ml. Worms were kept at 20 °C, and their lifespan was monitored. Worms feeding on bacteria carrying the empty vector or targeting *eri-1* were used as negative controls. At least 96 wells of the empty vector control were included. At the time when all of the control worms were dead, each well containing the different RNAi bacteria was scored for live worms. RNAi wells in which live worms were observed were scored as positives. RNAi clones that were scored as positive in the first and second passes of screening (Figure S1) were retested in duplicate during a third pass using a conventional longitudinal RNAi lifespan assays (see below).

### RNAi lifespan assay.

RNAi bacteria were prepared as described above. *daf-2, eri-1,* and *daf-16* RNAi clones were included as positive and negative controls in blind fashion in the conventional lifespan assays (*daf-2* RNAi construct kindly provided by M. Vidal, Harvard Medical School, Boston, Massachusetts, United States). Synchronous L4/young adult stage *eri-1(mg366)* animals treated with FUdR were prepared as above and placed on 6-well plates containing the positive RNAi clones and controls in duplicate. Approximately 40–60 adult animals were scored in each well (two wells for each RNAi clone, in two biological replicates). The animals were kept at 20 °C and scored every two days by gentle prodding with a platinum wire to test for viability. To ensure the continued efficacy of RNAi knockdown, animals were fed freshly induced RNAi bacteria every five to seven days. Lifespan is defined as the first day of adulthood (adult lifespan = 0) to when they were scored as dead. Worms that died of protruding/bursting vulva, bagging, or crawling off the agar were censored from the analysis. For epistasis analysis, RNAi lifespan assays were performed as described above, except that *daf-16(mgDf47);eri-1(mg366)* worms were used.

### Statistical analysis.

Statistical analyses were performed using SPSS software (http://www.spss.com). The survival experience of each RNAi-treated population is compared with that of the population treated with control RNAi using the log rank test. A *p*-value <0.05 was considered as significantly different from control.

### Arrested larvae survival.

Synchronized L1-stage *eri-1(mg366)* or *eri-1(mg366);daf-16(mgDf47)* animals were placed on RNAi clones that induce larval arrest phenotypes at 20 °C. To ensure that any animals bypassing lethality did not reproduce the plates were moved to 25 °C to exploit the temperature-sensitive sterility associated with *eri-1(mg366).* The wells were qualitatively monitored every two days for arrested larvae survival.

### Fluorescence microscopy assays.

Synchronized L1-stage animals or freshly egg-prepped embryos carrying the integrated transgenes *sod-3p::gfp, daf-16p::daf-16-gfp,* and *myo-3p::Q40yfp* were each placed onto RNAi bacteria as described above. The fluorescence intensity of each population was monitored two and three days following RNAi treatment.

### L4 fat accumulation.

Nile Red experiments were performed as previously described [66] except that L4-stage *eri-1(mg366)* animals were fed RNAi clones induced on plates containing 5 mM IPTG.

### Promoter fusions.

Promoter elements were amplified by PCR from wild-type genomic DNA and fused to RFP [67]. The transcriptional reporters were then injected into the gonads of wild-type adult hermaphrodites. Transgenic animals harboring the extrachromosomal arrays were then imaged.

### Primer pairs used to amplify promoter elements.

The primer pairs used to amplify promoter elements are as follows:

*c56g2.1*: F: 5′-CAGACAGGTGAAGCTGAGCGTGGC-3′, R: 5′-AAACGCAGAAAACGTCGGTGACGGAATG-3′;

*egl-45*: F: 5′-CCAGCCAGGAAAAATCGATTATATTAAG, R: 5′-AGTTGTGCTCGGATTACCGCTGAATTG-3′;

*unc-62*: F: 5′-CCCTGAAATTGTTGCGAAAGTTTCTG, R: 5′-GTTCCTGCAAGAGAGAAATATTAAATTTTG-3′;

*htp-3*: F: 5′-CTCCCGAAGATTCCGCATTTGCTC, R: 5′-TTTGACACTTAAAATATTTTAAAACATTTTTTTTAA-3′;

*f26a3.4*: F: 5′-GACCGGAACAGGTGGGCAATGTCGAC, R: 5′-TTTGTAGTGTATCTGTAATCATATTAAATTTGATTC-3′;

*inf-1*: F: 5′-GTATGTGTTTATGGTGTGTGCACAAG, R: 5′-GACAGGTGGGTTGAAAAGTTAAAAATTAAC-3′;

*f08b4.1*: F: 5′-CTAACCGATTCCTCAAGCCACGTGGG, R: 5′-TTTTGATTATTGATATTTCATTCGAATTTGCCAG-3′;

*y54e10br.4*: F: 5′-CTCTCCCGATTCCGCCATAATGCCCG, R: 5′-TCTCTGAAATATCGAAAAGAAATGAGATAATTG-3′;

*sem-5*: F: 5′-GGGTTAGAGCACTCTTAATGAGTCATG, R: 5′-CGTCTCGCTACCTGAAATATACTCTT-3′;

*zk686.2*: F: 5′-GGTTCCGGAGATAACCAAGCAGTATTGG, R: 5′-TATCTGGAGAAATTAAAATATGAACCAAAAAATGCG-3′.

## Supporting Information

Figure S1 A Subset of Longevity-Promoting RNAi Clones Are Expressed in Neuronal and Metabolic Tissues. (16.2 MB AI).

Figure S2 Candidate Longevity Genes Constitute Diverse but Novel Functional Groups The 64 RNAi clones identified are classified into broad functional groups: chaperones and protein turnover (6.3%); metabolism and mitochondria (15.6%); RNA, transcription factors, and nuclear chromatin (26.6%); signaling (21.9%); trafficking (4.7%); protein synthesis (15.6%); and unknown (9.4%).
(829 KB AI).

Figure S3 Survival Curves for Novel Classes of Lifespan Regulators Survival of *eri-1(mg366)* adult worms from the first day of adulthood (day 0) fed control vector (blue line), *daf-2*(RNAi) (red line), or RNAi to genes identified in our screen for increased mean lifespan. Representative genes from the protein synthesis and RNA and chromatin class are shown.
(958 KB AI).

Figure S4 Representative DAF-16-GFP Localization Phenotypes Under “normal” conditions DAF-16 is predominantly cytoplasmic (score = 0). Under conditions of stress DAF-16 is translocated from the cytoplasm to the nucleus (scores 1–3).
(3.7 MB AI).

Figure S5 Two-Hybrid Interaction Map of the 64-Candidate Longevity Genes Candidate genes identified are shown in red outline. This interaction map was generated using the Interactome query page from M. Vidal's research group (http://vidal.dfci.harvard.edu).
(284 KB AI).

Table S1 Phenotypes Associated with Candidate RNAi Clones Adult expression patterns identified in this study are highlighted in red and were determined as stated in the Materials and Methods. Developmental RNAi phenotypes and expression patterns of other genes were previously determined [40]. All, almost all cell types; C, coelomocytes; E, excretory cell; H, hypodermis; I, intestine; M, muscle; N, nervous system; P, pharynx; R, reproductive system; RG, renal gland; U, unidentified cell types. Homology is indicated by shading with BLASTP e-values <10^−6^ in related worm *(Cb),* yeast *(Sc),* fly *(Dm),* mouse (*Mn*)*,* and human (*Hs*). *sod-3p::gfp* expression was determined from four independent replicates. Adult fat accumulation was determined by Nile Red staining. Increased Nile Red staining is indicated by dark shading, and decreased Nile Red staining is indicated by light shading. Clones with decreased polyQ aggregation in adults compared to vector controls are indicated by shading.
(205 KB DOC)

Table S2 *daf-16(mgDf47);eri-1(mg366)* Lifespan Epistasis The mean lifespan is shown in days. The standard error of the mean lifespan is shown in days. The median lifespan and the time when 25% of individuals remain alive are shown in days. The total number of individuals scored is shown (followed by the number of individuals censored due to bursting, bagging, or crawling off the agar). The *p*-value from a log rank test comparing RNAi treatment populations to the control population is shown. Percent increase in mean lifespan of RNAi treated populations compared to the population fed vector control is shown.
(167 KB DOC)

## Abbreviations

CR: caloric restriction
RNAi: RNA interference
