# Supplementary material for: Lifespan Regulation by Evolutionarily Conserved Genes Essential for Viability
Source: PLoS Genet. 2007 Apr 6;3(4):e56. doi: 10.1371/journal.pgen.0030056 (PMC1847696; doi:10.1371/journal.pgen.0030056)
Supplement: Supplementary file 3 [file pgen.0030056.sg003.pdf]

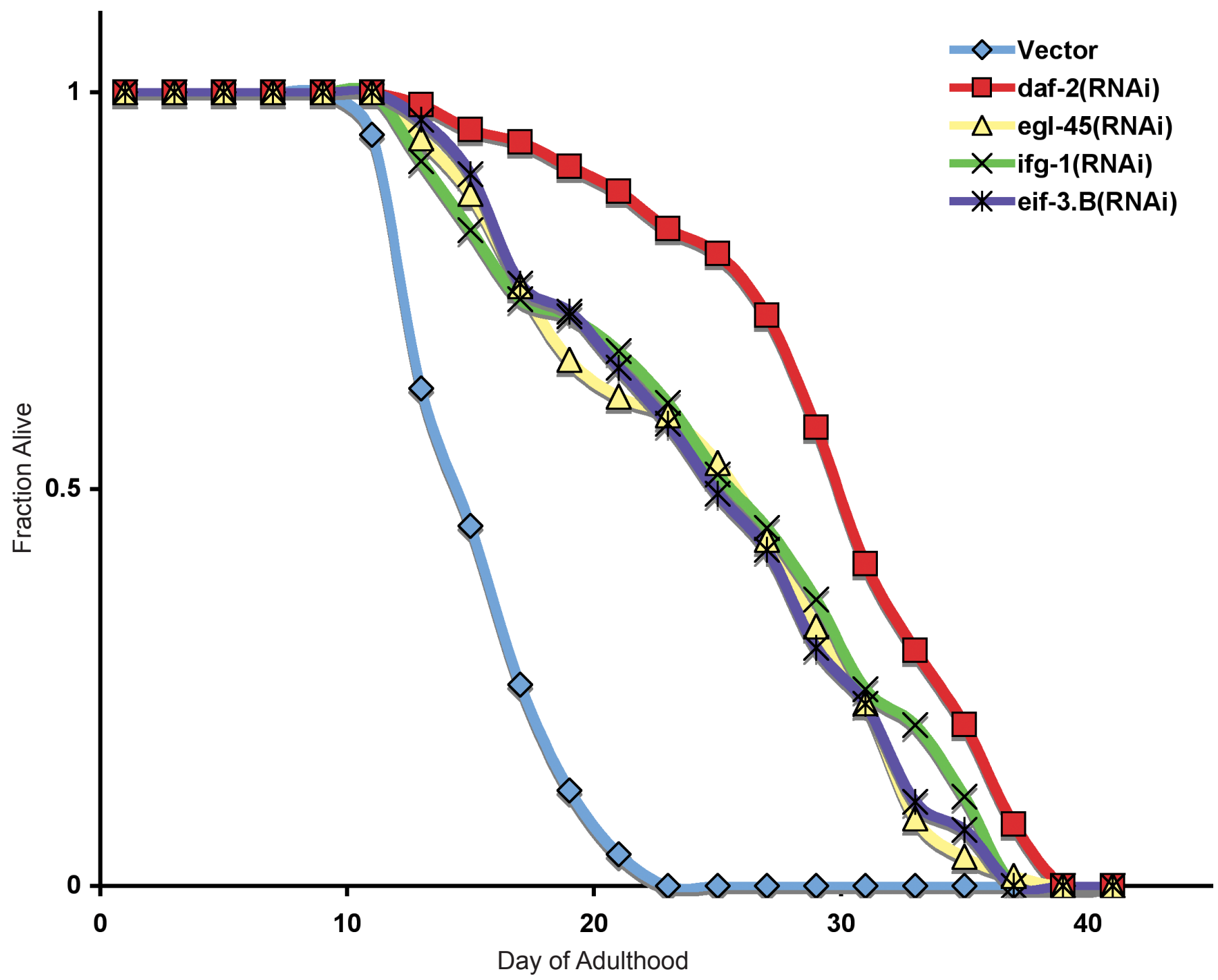

Protein Synthesis

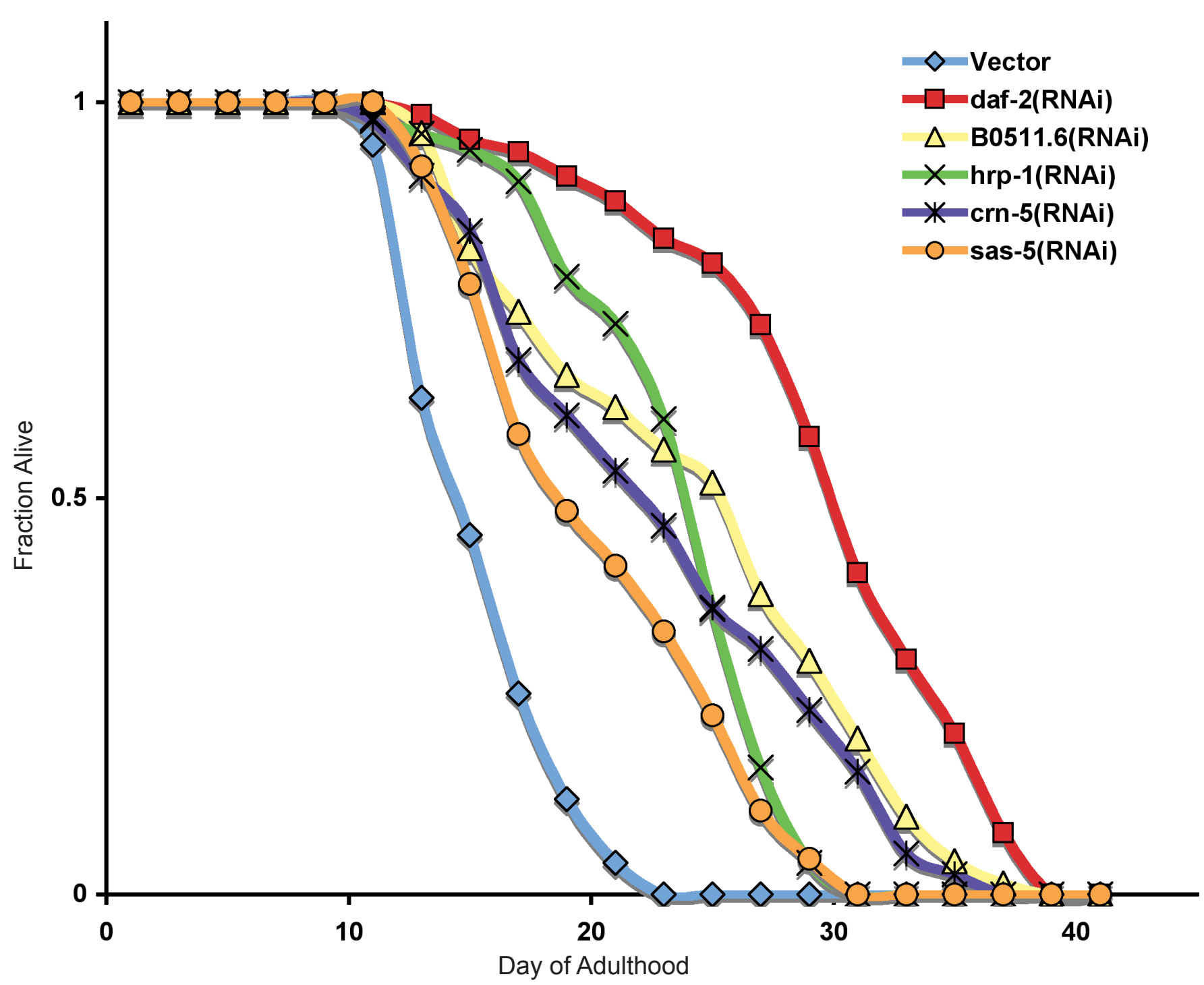

RNA and Chromatin

Supplemental Figure 3
Curran et. al.
