# Supplementary material for: Lifespan Regulation by Evolutionarily Conserved Genes Essential for Viability
Source: PLoS Genet. 2007 Apr 6;3(4):e56. doi: 10.1371/journal.pgen.0030056 (PMC1847696; doi:10.1371/journal.pgen.0030056)
Supplement: Supplementary file 6 [file pgen.0030056.st001.doc]

Supplemental Table 1. Phenotypes associated with candidate longevity genes

|  |  |  |  | **Homolog** | | | | | **L4 Fat** | **polyQ** |
| --- | --- | --- | --- | --- | --- | --- | --- | --- | --- | --- |
| **Sequence**  **Name** | **Locus** |  | **Expression** | **Cb** | **Sc** | **Dm** | **Mm** | **Hs** |  |  |
|  |  | **Developmental RNAi phenotypes** |  |  |  |  |  |  |  |  |
| **Y55D5A.5** | ***daf-2*** | **Age, Daf** | **I,N** |  |  |  |  |  |  |  |
| **Vector Control** |  |  |  |  |  |  |  |  |  |  |
| **T07A9.5** | ***eri-1*** |  | **N,R** |  |  |  |  |  |  |  |
| **B0511.6** |  | **Gro, Emb, Let, Lvl, Lva, Bmd, Stp** | **P,I,H,U** |  |  |  |  |  |  |  |
| **B0545.1** | ***tpa-1*** | **Gro, Emb, let, Lvl, Lva, Bmd, Stp** | **P,R,M,N,U** |  |  |  |  |  |  |  |
| **C01G8.5** | ***erm-1*** | **Gro, Emb, Let, Lvl, Lva, Dpy, Stp** | **P,I,RG,E,N** |  |  |  |  |  |  |  |
| **C09H10.3** | ***nuo-1*** | **Gro, Emb, Let, Lva, Sma, Bmd, Stp** |  |  |  |  |  |  |  |  |
| **C14A4.5** | ***crn-5*** | **Gro, Emb, Let, Lva, Bmd, Pch, Stp** |  |  |  |  |  |  |  |  |
| **C14F5.5** | ***sem-5*** | **Lon, Gro, Emb, Let, Lvl, Bmd, Pvl, Stp** | **N,M,I** |  |  |  |  |  |  |  |
| **C23G10.3** | ***rps-3*** | **Sck, Emb, Let, Lvl, Stp** |  |  |  |  |  |  |  |  |
| **C27D11.1** | ***egl-45*** | **Sck, Emb, Let, Lva, Dpy, Egl, Stp** | **N,C** |  |  |  |  |  |  |  |
| **C29F9.7** | ***pat-4*** | **Emb, Let, Pat, Lvl, Lva, Age, Bmd, Prl, Stp** | **P,R,l,M,N** |  |  |  |  |  |  |  |
| **C34C6.6** | ***prx-5*** | **Gro, Emb, Let, Lva, Bmd, Clr, Fat-R** |  |  |  |  |  |  |  |  |
| **C34D4.7/.6/.16** | ***str-49*** | **Gro, Emb, Let, Lvl, Lva, Stp** |  |  |  |  |  |  |  |  |
| **C34E10.6** | ***atp-2*** | **Gro, Emb, Let, Lva, Stp** | **P,M,I,H,E,N,U** |  |  |  |  |  |  |  |
| **C48D1.2** | ***ced-3*** | **Emb, Let, Let, Bmd, Ced, Stp** |  |  |  |  |  |  |  |  |
| **C56G2.1** |  | **Gro, Emb, Let, Lvl, Lva, Bmd, Stp** | **I,N,C** |  |  |  |  |  |  |  |
| **D1054.14** |  | **Gro, Sck, Emb, Let, Lva, Bmd, Stp** |  |  |  |  |  |  |  |  |
| **D2013.7** | ***eif-3.F*** | **Gro, Emb, Let, Lva, Bmd, Dpy, Pvl, Stp** |  |  |  |  |  |  |  |  |
| **D2030.9** |  | **Ric, Gro, Emb, Let, Lvl, Adl, Let, Bmd, Egl, Stp** | **P,I,R,M,H,N,U** |  |  |  |  |  |  |  |
| **F01F1.8** | ***cct-6*** | **Sck, Emb, Let, Lvl, Lva, Emb, Pvl, Clr, Stp** |  |  |  |  |  |  |  |  |
| **F02A9.6** | ***glp-1*** | **Emb, Let, Bmd, Stp** | **R,H** |  |  |  |  |  |  |  |
| **F08B4.1** | ***dic-1*** | **Emb, Let, Lva, Bmd, Stp** | **N, M** |  |  |  |  |  |  |  |
| **F19B6.1** |  | **Pvl, Stp** | **P,R,l,M,H** |  |  |  |  |  |  |  |
| **F25G6.2** |  | **Emb, Let, Lva, Bmd, Stp** |  |  |  |  |  |  |  |  |
| **F26A3.4** |  | **Gro, Emb, Let, Lvl, Lva, Bmd, Stp** | **All** |  |  |  |  |  |  |  |
| **F26E4.6** |  | **Gro, Age, Emb, let, Lvl, Lva, Bmd, Stp** |  |  |  |  |  |  |  |  |
| **F27C1.7** | ***atp-3*** | **Emb, Let, Lva, Age, Stp** | **P,I,R,M,H,N** |  |  |  |  |  |  |  |
| **F35B12.5** | ***sas-5*** | **Emb, Let** | **R** |  |  |  |  |  |  |  |
| **F35G12.3** | ***sel-5*** | **Gro, Emb, Let, Lvl, Lva, Bdm, Stp** |  |  |  |  |  |  |  |  |
| **F40F11.1** | ***rps-11*** | **Sck, Emb, Let, Stp** |  |  |  |  |  |  |  |  |
| **F42A6.7** | ***hrp-1*** | **Gro, Sck, Emb, Let, Lva, Bmd, Stp** | **All** |  |  |  |  |  |  |  |
| **F42C5.8/.9** | ***rps-8*** | **Sck, Lva, Stp** |  |  |  |  |  |  |  |  |
| **F43E2.7** |  | **Gro, Emb, Let, Lva, Bmd, Clr, Stp** | **P,M,N** |  |  |  |  |  |  |  |
| **F52E1.1** | ***pos-1*** | **Emb, Let, Bmd, Stp** | **R** |  |  |  |  |  |  |  |
| **F53F4.11** |  | **Gro, Emb, Let, Lvl, Lva, Bmd, Pvl, Stp** |  |  |  |  |  |  |  |  |
| **F57B9.6/.3** | ***inf-1*** | **Emb, let, Lva, Bmd, Stp** | **N** |  |  |  |  |  |  |  |
| **F57B9.6/.3** | ***inf-1*** | **Emb, let, Lva, Bmd, Stp** | **N** |  |  |  |  |  |  |  |
| **F57C9.5** | ***htp-3*** | **Gro, Emb, Let, Lvl, Lva, Bmd, Stp, Him** | **I** |  |  |  |  |  |  |  |
| **F59C6.5** |  | **Emb, Let, Lva, Stp** |  |  |  |  |  |  |  |  |
| **H28O16.1** | ***phi-37*** | **Gro, Emb, Let, Lva, Stp** | **P,I,M,U** |  |  |  |  |  |  |  |
| **K01C8.10** | ***cct-4*** | **Rup, Emb, Let, Lva, Bmd, Pch, Stp** | **P,I,RG,U** |  |  |  |  |  |  |  |
| **M03A8.4** | ***gei-15*** | **Gro, Emb, Let, Lvl, Lva, Bmd, Stp** |  |  |  |  |  |  |  |  |
| **M110.4** | ***ifg-1*** | **Gro, Emb, Let, Lva, Bmd, Pch** | **I,P,N,R,M** |  |  |  |  |  |  |  |
| **T07A9.8** |  | **Gro, Emb, let, Bmd, Pch, Stp** |  |  |  |  |  |  |  |  |
| **T20F10.1** | ***tag-181*** | **Gro, Emb, Let, Lvl, Lva, Bmd, Slu, Stp** | **P,M,R,U** |  |  |  |  |  |  |  |
| **T21B10.1** |  | **Gro, Emb, Let, Lva, Sma, Bmd, Clr, Stp** |  |  |  |  |  |  |  |  |
| **T27F7.3** |  | **Gro, Emb, Let, Lvl, Lva, Bmd, Dpy, Pch, Stp** | **P,I,RG,N** |  |  |  |  |  |  |  |
| **T28F12.2** | ***unc-62*** | **Emb, Let, Bmd, Dpy, Mig, Egl, Pvl, Stp** | **N** |  |  |  |  |  |  |  |
| **VC5.3** | ***npa-1*** | **Gro, Emb, Let, Lvl, Lva, Bmd, Muv, Stp** |  |  |  |  |  |  |  |  |
| **VW02B12L.1** | ***vha-6*** | **Emb, Let, Lva, Bmd, Dpy, Clr, Stp** | **I,H,R** |  |  |  |  |  |  |  |
| **W07E6.1** |  | **Lon, Gro, Sle, Emb, Lva, Let, Bmd, Pch, Stp** | **I,M,N,H,R** |  |  |  |  |  |  |  |
| **Y105C5B.12/C06E7.1** |  | **Gro, Emb, Let, Lvl, Lva, Bmd, Slu, Pvl, Stp, Fat** |  |  |  |  |  |  |  |  |
| **Y105E8B.8** | ***ero-1*** | **Emb, Let, Lva, Bmd, Stp** |  |  |  |  |  |  |  |  |
| **Y110A7A.12/F20B6.2** | ***tag-300*** | **Gro, Emb, Let, Lvl, Lva, Adl, Bmd, Prl, Stp** |  |  |  |  |  |  |  |  |
| **Y17G7B.5** | ***mcm-2*** | **Emb, Let, Pvl, Stp** | **I,N** |  |  |  |  |  |  |  |
| **Y47G6A.10** | ***spg-7*** | **Gro, Emb, Let, Lva, Bmd, Stp** |  |  |  |  |  |  |  |  |
| **Y54E10BR.4** |  | **Gro, Sck, Emb, Let, Lva, Bmd, Pvl, Stp** | **N, M** |  |  |  |  |  |  |  |
| **Y54E2A.11** | ***eif-3.B*** | **Gro, Emb, Let, Lvl, Lva, Bmd, Stp** | **P,M,R,M,N,U** |  |  |  |  |  |  |  |
| **Y56A3A.19** |  | **Gro, Emb, Let, Lva, Bmd, Stp** | **P** |  |  |  |  |  |  |  |
| **Y57A10C.5** | ***sre-25*** | **Gro, Emb, Let, Lvl, Lva, Bmd, Stp** | **I,N** |  |  |  |  |  |  |  |
| **Y65B4BR.5** |  | **Gro, Emb, Let, Lva, Bmd, Dpy, Mlt, Pvl, Stp, Him** |  |  |  |  |  |  |  |  |
| **ZC101.2** | ***unc-52*** | **Gro, Emb, Let, Lvl, Adl, Prl** | **P,I,R,l,M,H** |  |  |  |  |  |  |  |
| **ZC132.3** |  | **Gro, Emb, Let, Lvl, Lva, Bmd, Stp** |  |  |  |  |  |  |  |  |
| **ZK1127.5** |  | **Gro, Emb, Let, Lvl, Lva, Bmd, Dpy, Egl, Prl, Pvl, Pch, Stp** | **I,RG** |  |  |  |  |  |  |  |
| **ZK686.2** |  | **Gro, Emb, Let, Lva, Bmd, Stp** | **I, N** |  |  |  |  |  |  |  |
| **ZK792.3/.2** | ***inx-9*** | **Emb, Let, Bmd, Stp** | **N,R** |  |  |  |  |  |  |  |
