# Supplementary material for: Lifespan Regulation by Evolutionarily Conserved Genes Essential for Viability
Source: PLoS Genet. 2007 Apr 6;3(4):e56. doi: 10.1371/journal.pgen.0030056 (PMC1847696; doi:10.1371/journal.pgen.0030056)
Supplement: Supplementary file 7 [file pgen.0030056.st002.doc]

**Supplemental Table 2 – *daf-16(mgDf47);eri-1(mg366)*** lifespan epistasis

| **Sequence**  **Name** | **Mean** | **Std.**  **Err** | **Med/**  **25%** | **Total/**  **Cens** | **p-value** | **% Extension** |
| --- | --- | --- | --- | --- | --- | --- |
| Y55D5A.5 | **9.51** | **0.19** | **9/11** | **99/3** | **0.05** | -5.9 |
| L4440 | **10.11** | **0.19** | **11/11** | **95/1** | **NA** | 0.0 |
| T07A9.5 | **9.33** | **0.22** | **9/11** | **80/5** | **0.4817** | -7.7 |
| F27C1.7 | **12.92** | **0.37** | **13/15** | **92/1** | **<.00001** | 27.8 |
| F59C6.5 | **12.11** | **0.32** | **13/13** | **80/1** | **<.00001** | 19.8 |
| H28O16.1 | **12.57** | **0.3** | **13/15** | **114/2** | **<.00001** | 24.3 |
| Y47G6A.10 | **9.74** | **0.16** | **9/11** | **99/2** | **0.7487** | -3.7 |
| T27F7.3 | **11.28** | **0.24** | **11/13** | **81/3** | **<.00001** | 11.6 |
| F43E2.7 | **13.2** | **0.3** | **13/15** | **120/0** | **<.00001** | 30.6 |
| M110.4 | **14.45** | **0.33** | **15/17** | **97/3** | **<.00001** | 42.9 |
| K01C8.10 | **11.72** | **0.2** | **11/13** | **75/9** | **<.00001** | 15.9 |
| VW02B12L.1 | **11.88** | **0.27** | **11/13** | **79/7** | **<.00001** | 17.5 |
| C09H10.3 | **10.01** | **0.21** | **11/11** | **74/8** | **0.1264** | -1.0 |
| Y17G7B.5 | **10.33** | **0.23** | **11/11** | **107/4** | **0.0111** | 2.2 |
| ZC101.2 | **9.68** | **0.2** | **9/11** | **66/1** | **0.9309** | -4.3 |
| C29F9.7 | **12.77** | **0.3** | **13/13** | **103/2** | **<.00001** | 26.3 |
| Y65B4BR.5 | **10.18** | **0.17** | **11/11** | **84/1** | **0.0609** | 0.7 |
| C56G2.1 | **9.86** | **0.22** | **9/11** | **50/1** | **0.563** | -2.5 |
| F01F1.8 | **10.68** | **0.15** | **11/11** | **117/6** | **<.00001** | 5.6 |
| F02A9.6 | **9.68** | **0.2** | **9/11** | **99/10** | **0.6461** | -4.3 |
| F08B4.1 | **13.47** | **0.4** | **13/15** | **73/1** | **<.00001** | 33.2 |
| ZK792.3 | **10.15** | **0.21** | **11/11** | **107/7** | **0.037** | 0.4 |
| T28F12.2 | **13.23** | **0.28** | **11/13** | **71/6** | **0.0197** | 30.9 |
| F52E1.1 | **9.7** | **0.19** | **9/11** | **86/4** | **0.762** | -4.1 |
| F25G6.2 | **11.12** | **0.18** | **11/13** | **118/4** | **<.00001** | 10.0 |
| D1054.14 | **10.83** | **0.18** | **11/11** | **108/4** | **<.00001** | 7.1 |
| F35B12.5 | **9.62** | **0.18** | **9/11** | **81/4** | **0.9197** | -4.8 |
| C14F5.5 | **10.19** | **0.18** | **9/11** | **108/6** | **0.0241** | 0.8 |
| F57C9.5 | **11.11** | **0.14** | **11/11** | **118/52** | **<.00001** | 9.9 |
| Y110A7A.12 | **9.28** | **0.13** | **9/11** | **137/10** | **0.1329** | -8.2 |
| C01G8.5 | **9.63** | **0.17** | **9/11** | **101/2** | **0.987** | -4.7 |
| D2030.9 | **10.38** | **0.13** | **11/11** | **94/25** | **0.0058** | 2.7 |
| F26A3.4 | **9.86** | **0.19** | **9/11** | **113/11** | **0.3407** | -2.5 |
| F26E4.6 | **12.24** | **0.28** | **13/13** | **113/4** | **<.00001** | 21.1 |
| T20F10.1 | **9.4** | **0.18** | **9/11** | **92/11** | **0.4009** | -7.0 |
| B0511.6 | **11.24** | **0.25** | **11/13** | **105/8** | **<.00001** | 11.2 |
| Y54E10BR.4 | **10.01** | **0.17** | **11/11** | **90/7** | **0.179** | -1.0 |
| W07E6.1 | **9.77** | **0.16** | **9/11** | **76/5** | **0.8232** | -3.4 |
| C34C6.6 | **9.89** | **0.21** | **11/11** | **97/3** | **0.1938** | -2.2 |
| C14A4.5 | **9.74** | **0.2** | **9/11** | **78/3** | **0.6438** | -3.7 |
| Y57A10C.5 | **9.97** | **0.21** | **11/11** | **74/2** | **0.2178** | -1.4 |
| Y54E2A.11 | **14.02** | **0.27** | **13/15** | **113/3** | **<.00001** | 38.7 |
| C34E10.6 | **10.17** | **0.14** | **11/11** | **85/3** | **0.066** | 0.6 |
| F57B9.6 | **12.9** | **0.36** | **13/15** | **102/2** | **<.00001** | 27.6 |
| F57B9.6 | **11.29** | **0.33** | **11/13** | **85/1** | **<.00001** | 11.7 |
| ZK686.2 | **9.74** | **0.2** | **9/11** | **73/5** | **0.6792** | -3.7 |
| C27D11.1 | **13.33** | **0.39** | **13/15** | **82/7** | **<.00001** | 31.8 |
| Y56A3A.19 | **9.41** | **0.26** | **9/11** | **50/1** | **0.5984** | -6.9 |
| T07A9.8 | **9.85** | **0.29** | **9/11** | **51/5** | **0.3981** | -2.6 |
| F42A6.7 | **10.91** | **0.29** | **11/11** | **46/4** | **0.0006** | 7.9 |
| B0545.1 | **9.51** | **0.17** | **9/11** | **76/6** | **0.5386** | -5.9 |
| F42C5.8 | **12.63** | **0.45** | **11/15** | **65/1** | **<.00001** | 24.9 |
| ZC132.3 | **10.04** | **0.3** | **9/11** | **56/7** | **0.1365** | -0.7 |
| VC5.3 | **9.85** | **0.16** | **9/11** | **114/16** | **0.4277** | -2.6 |
| C23G10.3 | **12.45** | **0.37** | **13/15** | **87/7** | **<.00001** | 23.1 |
| F53F4.11 | **9.94** | **0.18** | **9/11** | **117/2** | **0.1748** | -1.7 |
| F19B6.1 | **10.23** | **0.16** | **11/11** | **85/4** | **0.0443** | 1.2 |
| C48D1.2 | **10.21** | **0.18** | **11/11** | **69/3** | **0.0641** | 1.0 |
| Y105C5B.12 | **10.28** | **0.17** | **11/11** | **83/2** | **0.0206** | 1.7 |
| Y105E8B.8 | **9.2** | **0.21** | **9/11** | **54/2** | **0.1401** | -9.0 |
| ZK1127.5 | **10.22** | **0.2** | **9/11** | **77/4** | **0.0426** | 1.1 |
| T21B10.1 | **9.96** | **0.18** | **11/11** | **75/7** | **0.318** | -1.5 |
| D2013.7 | **14.02** | **0.35** | **15/17** | **104/6** | **<.00001** | 38.7 |
| F35G12.3 | **10.21** | **0.21** | **11/11** | **71/3** | **0.0506** | 1.0 |
| F40F11.1 | **13.58** | **0.31** | **13/15** | **103/6** | **<.00001** | 34.3 |
| C34D4.7 | **10.49** | **0.22** | **11/11** | **79/6** | **0.003** | 3.8 |
| M03A8.4 | **10.14** | **0.27** | **9/11** | **67/2** | **0.0659** | 0.3 |
